# A Novel Approach of Preventing Japanese Cedar Pollen Dispersal That Is the Cause of Japanese Cedar Pollinosis (JCP) Using Pollen-Specific Fungal Infection

**DOI:** 10.1371/journal.pone.0062875

**Published:** 2013-05-07

**Authors:** Yuuri Hirooka, Mitsuteru Akiba, Yu Ichihara, Hayato Masuya, Yoshihiro Takahata, Tomohisa Suda, Yutaka Yada, Shigehiro Yamamoto, Takanori Kubono

**Affiliations:** 1 Department of Forest Microbiology, Forestry and Forest Products Research Institute, Tsukuba, Ibaraki, Japan; 2 Tohoku Research Center, Forestry and Forest Products Research Institute, Morioka, Iwate, Japan; 3 Kyushu Research Center, Forestry and Forest Products Research Institute, Kurokami, Kumamoto, Japan; 4 Fukushima Prefectural Forestry Research Center, Koriyama, Fukushima, Japan; 5 Ishikawa Forest Experiment Station, Hakusan, Ishikawa, Japan; 6 Shizuoka Prefectural Research Institute of Agriculture and Forestry, Forestry and Forest Products Research Center, Hamamatsu, Shizuoka, Japan; California Department of Public Health, United States of America

## Abstract

In Japan, Japanese cedar pollen dispersal is one of the major causes of pollinosis. *Sydowia japonica* is an ascomycetous fungus that grows exclusively on the male strobili of Japanese cedar, suggesting a possible mechanism for controlling pollen dispersal. To evaluate this possibility, eleven isolates of *S. japonica* were collected from around Japan and used as an inoculum to male strobili of Japanese cedar. The treatment demonstrated that the fungus infected only the pollen and prevented pollen dispersal. The fungus did not cause any additional symptoms to other parts of Japanese cedar, such as needles, stems, and buds. All *S. japonica* isolates collected around Japan could serve to control pollen dispersal. Periodic observation of the fungal pathogenesis with stereomicroscope and scanning electron microscope showed that hyphal fragments and conidia of *S. japonica* germinated on the surface of male strobili, and the germ tube entered pollen sacs through opening microsporophylls. Within the pollen sacs, the hyphae penetrated pollen gradually, such that all pollen was infected by the fungus by approximately one month before the pollen dispersal season. The infected pollen was destroyed due to the fungal infection and was never released. Our data suggests a novel approach of preventing pollen dispersal using pollen-specific fungal infection.

## Introduction

Japanese cedar (*Cryptomeria japonica* D. Don), which has a natural distribution between Mt. Yagura, Aomori prefecture (40° 42′ N) and Yaku Island, Kagoshima prefecture (30°15′ N) [1], is the most important coniferous tree in Japan. Artificial planting of Japanese cedar was brought into practice more than 500 years ago and conducted intensively after World WarII [2]. In recent years, it has been estimated that the plantings of this tree cover 40% of the total cultivated area of Japan, representing 12% of Japanese forests [3], [4]. Historically, Japanese cedar was commonly incorporated into the landscape of temples and shrines as a sacred tree. Since Japanese cedar is cultivated for many years and regularly releases large amounts of pollen every spring, Japanese cedar pollinosis (JCP) has become the most common type of allergic rhinitis in Japan. A recent nationwide epidemiological survey found that at least 25% of the Japanese population suffers from JCP [5]. During the pollen dispersal season, many Japanese people who suffer JCP opt to wear glasses and a mask to avoid pollen exposure, or to take chemical agents to ameliorate their allergic symptoms. Two pollen proteins, Cry j 1 and Cry j 2, are thought to be important allergens in the pathogenesis of JCP [6]–[8]. Although many studies on JCP have been conducted since the remarkable increase in pollinosis frequency in the 1970s, the number of JCP patients continues to rise each year [9].

One counter measure to JCP is the reduction of the amount of pollen released into the environment by Japanese cedar forests. In recent years, the use of male-sterile Japanese cedars that release no pollen has been proposed. In 1992, the first natural male-sterile Japanese cedar was found in Toyama prefecture, Japan [10], and subsequently more than 24 separate genotypes of male-sterile Japanese cedar have been found in several parts of Japan [11]. At the same time, researchers have developed a micropropagation technique that uses an *in vitro* culture of male-sterile Japanese cedar genotypes to develop ideal male-sterile Japanese cedar trees [12], [13]. Recently, genomic approaches have identified many nonredundant full-length enriched cDNA clones of male-sterile Japanese cedar, and molecular genetic analysis is being used to clarify the mechanisms of development of male reproductive organs [14]–[17]. However, replacement of male-fertile with male-sterile Japanese cedar is a time-consuming and tedious process. Others studies have investigated approaches with more immediate effects. For instance, the herbicide compounds maleic hydrazide and trinexapac ethyl have been used to suppress male flower formation [18], [19]. However, it would not be feasible to spray such chemicals at temples and shrines, where as mentioned above Japanese cedars are commonly planted and many people gather. To identify less toxic alternatives, Koshio et al. [20], [21] examined the effect on male strobili of Japanese cedar of oleic acid and nonionic surfactants formulated from oleic acid derivatives. However, testing using a range of cedar clones and exposures during periods of male flower initiation failed to demonstrate significant effects on pollen production. In addition, such treatments could affect non-target species of plants in the natural areas, because the oleic acid derivatives can inhibit ethylene evolution. Therefore, novel treatments that could selectively inhibit the release of Japanese cedar pollen remain to be identified.

In 2005, in the Fukushima prefecture of Japan, we discovered many Japanese cedar male strobili infected by a peculiar ascomycetous fungus. Subsequent work revealed that this fungus had been described previously by Kasai [22] as *Leptosphaerulina japonica* Kasai, and had been reported only a few times since then [23], [24]. Hirooka et al. [25] reported a taxonomic re-appraisal with newly collected specimens from various parts of Japan, and the fungus now has been transferred to the genus *Sydowia* (Dothioraceae, Dothideales, Dothideomycetes, Ascomycota) as *Sydowia japonica* (Kasai) Hirooka & Masuya based on morphological and molecular data. Interestingly, this fungal infection occurred only on male strobili including abundant pollen, and was detected even after strict surface sterilization of the male strobili. Additionally, our preliminary observations indicated that abundant fungal mycelia were observed around undispersed pollen that was not able to be emitted during the pollen dispersal season. We hypothesized that this fungus is parasitic only on Japanese cedar pollen and could serve as a biocontrol agent for controlling pollen dispersal. To evaluate this possibility, male strobili were treated with any of eleven isolates of *S. japonica* and then assessed for pollen dispersal. In addition, the parasitic behavior of *S. japonica* was observed using the stereomicroscope and scanning electron microscope (SEM) to better understand the pathology of the fungus when infecting pollen.

## Materials and Methods

### Field Survey and Preservation of Specimens

From 2006 to 2012, male strobili of Japanese cedar that had been infected by *Sydowia japonica* were collected by the authors or collaborating scientists. In case of that *S*. *japonica* has a broad host range, we sought the fungus on male flowers of other plant especially gymnosperm species. No specific permits were required for the described field studies. From each specimen, a few fungal bodies with infected male strobili were removed and air-dried for later isolation. The rest of the specimens were kept in a low humidity room and deposited in the Herbaria of Forest Mycology and Pathology (TFM), Forestry and Forest Products Research Institute, Tsukuba, Ibaraki, Japan.

### Fungal Cultures and Isolation

Cultures were obtained directly from the tissues of infected male strobili or from spores. For culturing from strobili, the surfaces of male strobili were sterilized by sequential immersion in 80% ethanol for 30 sec and 0.1% mercuric chloride for 30 sec, rinsed three times with sterilized water, and then dried on sterilized filter paper. The treated male strobili were cultured directly on 2% malt agar (MA; malt extract 20 g, agar 15 g, distilled water 1000 mL), and emerging hyphae were transferred to slants of Difco™ potato dextrose agar (PDA; Detroit, Michigan, USA) and incubated at 20°C. Also single hyphae directly growing on male strobili were transferred to 2% malt agar with a tungsten needle (Nissin EM Co., Tokyo, Japan) or a fine insect pin to confirm that hyphae belonged to *S. japonica*. For culturing from spores, fungal fructifications on infected male strobili were crushed in water, and the resulting ascospore suspension was streaked on 2% water agar (WA) and incubated at 20°C. After 24 h, individual germinating or budding spores were transferred directly to PDA slants.

To identify *S*. *japonica* from all isolated cultures, morphological characters according to Hirooka et al. [25] were used.

Newly isolated cultures were preserved in the culture collection of the Forestry and Forest Products Research Institute (FFPRI; Ibaraki, Japan). Isolates of *S. japonica* previously preserved in culture collections of the FFPRI or Biological Resource Center (NITE; Chiba, Japan) also were obtained and examined in this study.

### Microsporogenesis of Japanese Cedar Pollen

The pollen microspore stage of Japanese cedar was determined by observing the inside of male strobili every 7 days from September until the pollen dispersal season started, in the field facilities of FFPRI, Tukuba, Japan (36°0′36″N, 140°7′33″E). A fluorescent microscope (Nikon Model, Eclipse 80i, Tokyo, Japan) was used to observe the pollen development.

### Colony Growth and Temperature

Disks of 3 mm diameter were cut from the edges of young colonies and placed in the center of MA plates, which then were incubated at temperatures from 5 to 35°C at 5°C intervals in complete darkness. The diameters of the colonies on five plates for each isolate at each temperature were tracked for two weeks. This trial was replicated five times for each isolate, and standard error was computed using Systat 10.2 (Systat Software, San José, California, USA).

### Field Tests of Pollen Dispersal Reduction Following Treatment with S. japonica

The *in situ* ability of *S. japonica* isolates to reduce pollen dispersal was assessed by applying fungal hyphal fragments and conidia to male strobili in the three field facilities of FFPRI in Tsukuba, Japan. The field trials were performed as separate experiments during the 2010–2011 and 2011–2012 field seasons. The two field facilities: field A and B were used during the 2010–2011 seasons while field C was used during the 2011–2012 seasons. Before the inoculation tests, the formation of male strobili was induced by 100 ppm gibberellin (Kyowa-Hakko, Tokyo, Japan) to about 20 year old Japanese cedar trees. Eleven isolates of *S. japonica* collected around Japan were used as the inocula (Table 1). For each fungal isolate, the inoculum was prepared by growing cultures in rice bran/wheat bran/water medium (1∶1:1) (RWBM) for two weeks at 18°C. In the field, up to 20 male strobili were directly attached with each inoculum that was cut out as blocks of about 5 mm^3^ and then bound with vinyl tape. As controls, other male strobili were treated with RWBM lacking the fungus. After 14 days, the vinyl tape was removed from the male strobili. The treatments were carried out three times in total: twice at the beginning of November in 2010 and once at the beginning of November in 2011, when Japanese cedar pollen becomes mature and *S. japonica* usually produced abundant spores. The treated male strobili were observed every 7 days until May of the following year that is until the end of the pollen dispersal season. Efficacy of prevention of pollen dispersal was defined by number of male strobili including the infected pollen that are completely packed by abundant hyphae of *S. japonica* after the pollen dispersal season. Significant differences among treatment frequency (at P = 0.05) of the data were compared with Ryan’s test [26].

**Table 1:** *Sydowia japonica* isolates used in this study.

Isolate No.	Location, prefecture	Collection date	Origin
FFPRI 411102	Nishimeya, Aomori	July, 2010	Tissue of an infected male strobilus
FFPRI 411103	Akita, Akita	June, 2009	Tissue of an infected male strobilus
FFPRI 411104	Shizukuishi, Iwate	July, 2010	Tissue of an infected male strobilus
NITE P-757	Yama-gun, Fukushima	September, 2007	Ascospore
FFPRI 411105	Hamamatsu, Shizuoka	June, 2010	Tissue of an infected male strobilus
FFPRI 411084	Nakanikawa-gun, Toyama	December, 2010	Tissue of an infected male strobilus
FFPRI 411087	Hakui-gun, Ishikawa	September, 2010	Tissue of an infected male strobilus
FFPRI 411106	Otsu, Shiga	February, 2011	Tissue of an infected male strobilus
FFPRI 411107	Iinan, Shimane	November, 2010	Tissue of an infected male strobilus
FFPRI 411108	Iyomishima, Ehime	March, 2011	Tissue of an infected male strobilus
FFPRI 411109	Ituki-mura, Kumamoto	October, 2010	Tissue of an infected male strobilus

**FFPRI**: Forestry and Forest Products Research Institute, Ibaraki, Japan; **NITE**: Biological Resource Center, Chiba, Japan.

### Behavior of S. japonica on Male Strobili as Observed with Stereomicroscope and SEM

All treated male strobili were divided vertically with a razor before observing. One side was used for permitting observation of the interior infected pollen, and another side was used to isolate *S. japonica* by way of precaution. Macroscopic observations were made by using a stereomicroscope (Nikon SMZ-1500, Tokyo, Japan). For SEM observation, treated and untreated male strobili were fixed overnight in 5% glutaraldehyde in 0.1 M phosphate buffer (pH 7.2), then post-fixed in 0.2 M osmium tetroxide for 90 min, dehydrated in a series of ethanol solutions, and freeze-dried in a dessication device (JFD-320, JEOL, Tokyo). Each male strobilus was coated with Pt-Pd (80∶20) in an ion-sputter coater for 5 min at the condition of 1.5 KeV and 15 mA. An SEM (S-4800 FE-SEM, Tokyo) operating at 1 KV was used to examine the specimens.

## Results

### Field Survey

During intensive collecting around Japan for seven years, we isolated *S*. *japonica* only from male strobili of Japanese cedar. Other plants infected by *S*. *japonica* have never been observed in nature. Total eleven isolates were collected between Nishimeya, Aomori prefecture (40° 34′ N) and Ituki-mura, Kumamoto prefecture (32°15′ N) (Table 1).

### Fungal Cultures and Isolation

Among 150 isolates obtained from infected male strobili, twelve species were identified as *Alternaria* sp., *Clonostachys rosea* (Link) Schroers, Samuels, Seifert & W. Gams, *Cladosporium cladosporioides* (Fresen) G.A. de Vries, *Cladosporium* sp., *Cylindrosympodium* sp., *Endoconidioma* sp., *Fulvoflamma* sp., *Fusarium ciliatum* Link, *Pestalotiopsis* sp., *Trichoderma* sp., *Sydowia japonica*, and *Zalerion* sp. Among these isolates, *S*. *japonica* has the highest detection rate (80%).

### Microsporogenesis of Japanese Cedar Pollen

**Figure 1:**
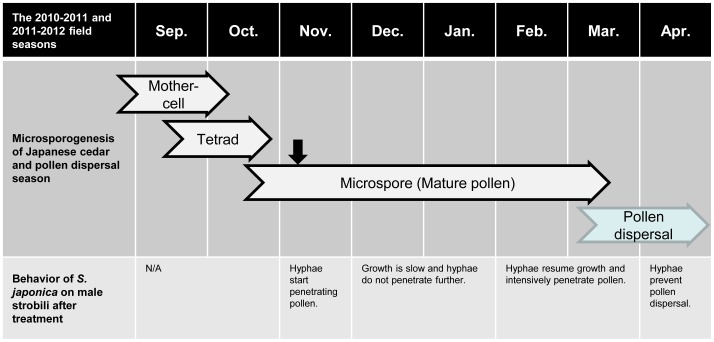
Diagram of microsporogenesis of Japanese cedar and behavior of *S. japonica* on male strobili in each month until the end of pollen dispersal season in the field facilities of FFPRI in Tsukuba, Japan. Black arrow indicates the treatment date.

Pollen mother-cell and pollen tetrad were found until October, and then the mature pollen in microspore stage was gradually observed from the end of October in the field. Although all mature pollen appeared in a pollen sac by the beginning of November, the pollen was not released until the beginning of the pollen dispersal season (Fig. 1). Therefore, our treatments were carried out soon after observing the mature pollen.

### Colony Growth and Temperature

**Figure 2:**
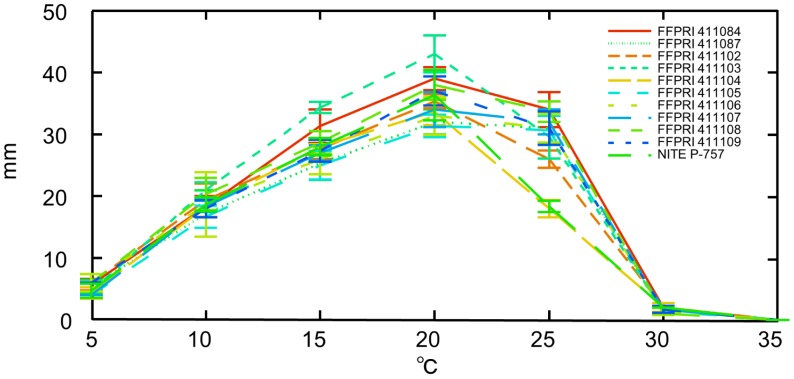
Mycelial growth of *Sydowia japonica*. Colony diameters of seven isolates were measured after two weeks’ growth on 2% MA at the indicated temperatures. Error bars represent ± standard error.

Isolates collected from various sites around Japan exhibited comparable growth after two weeks. All grew at temperatures from 5–30°C, although growth was minimal at the upper end of the range; optimal growth was observed at a temperature of 20°C (Fig. 2). On MA, all isolates initially grew as white colonies, slowly turning gray in color before further darkening.

### Field Tests of Pollen Dispersal Reduction Following Treatment with S. japonica

**Figure 3:**
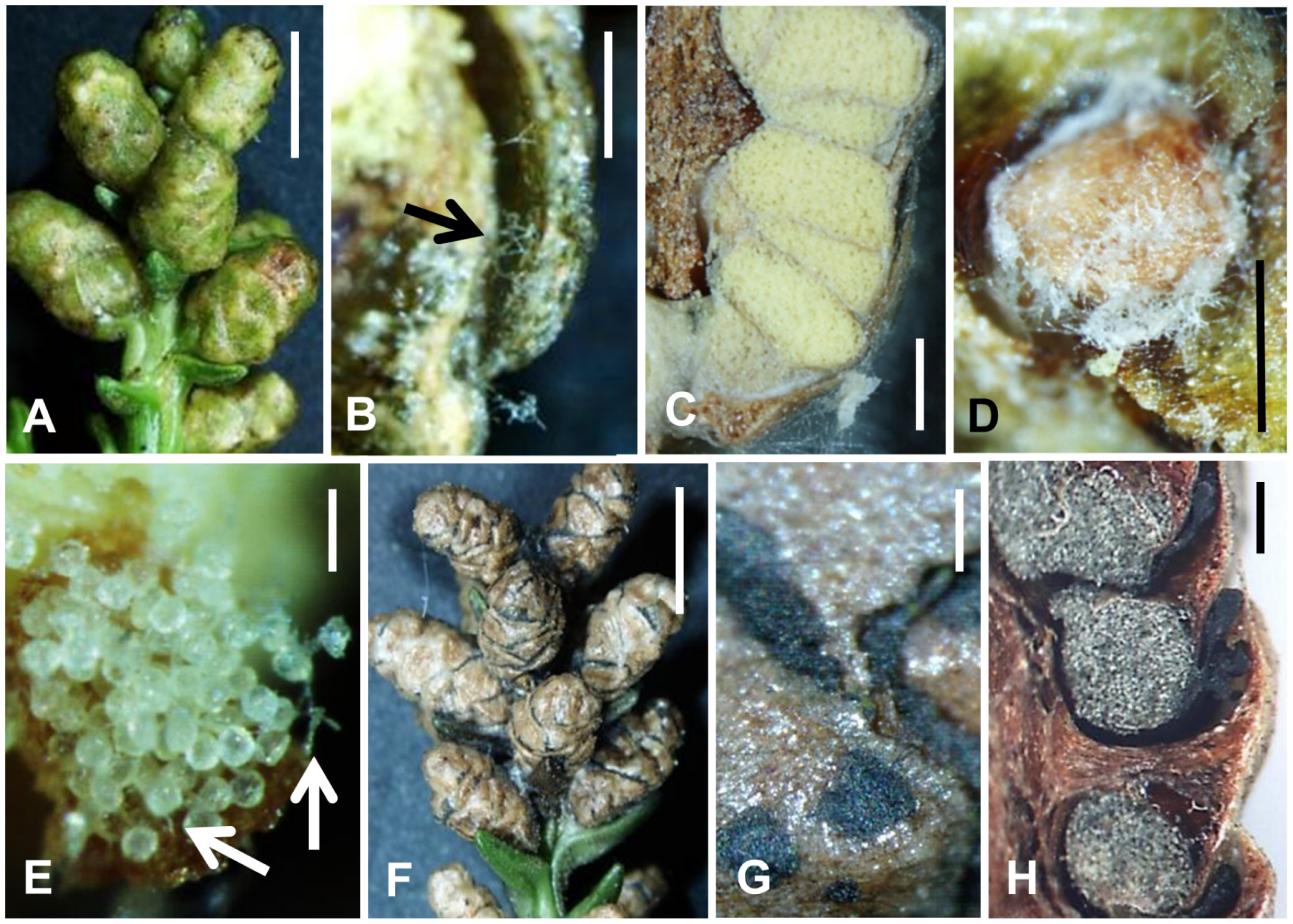
Stereomicrographs of male strobili of Japanese cedar treated with *Sydowia japonica*. A. Treated Japanese cedar male strobili, about four months after treatment (middle of March, 2012) (bar = 5 mm). B. Hyphae under a microsporophyll (arrow), about four months after treatment (bar = 100 µm). C. Vertical section of a treated male strobilus and white hyphae of *S. japonica* around each pollen sac, about four months after treatment (bar = 500 µm). D. White hyphae of *S. japonica* around a pollen sac, about four months after treatment (bar = 500 µm). E. Pollen infected by hyphae of *S. japonica* (arrows), about four months after treatment (bar = 50 µm). F. Treated Japanese cedar male strobili, about six months after treatment (end of April, 2012) (bar = 5 mm). G. Conidial fructifications, acervuli, of *S. japonica* on a treated male strobilus, about six months after treatment (bar = 100 µm). H. Vertical section of a treated male strobilus and black stromata of *S. japonica* around each pollen sacs, about six months after treatment (bar = 200 µm).

On average, treatment with *S. japonica* in the field prevented pollen dispersal in more than 50% of the treated male strobili, although the prevention frequency varied somewhat between the 2010 and 2011 field seasons (Table 2). Notably, isolate obtained from Kumamoto (FFPRI 411109) was statistically distinguishable from control and included in the high prevention category at the all fields during the 2010–2011 and 2011–2012 seasons. In addition, the isolate provided more than 80% prevention in total. Although treated and control male strobili were indistinguishable at the beginning of the season, pollen was never emitted from the treated strobili even when the strobili were strongly shaken (Fig. 3 A). Within treated male strobili, abundant pollen sacs and pollen infected by slightly melanized hyphae were found at the beginning to middle of the season (Fig. 3 B–E). The remaining pollen in these male strobili was gradually becoming gray due to the infection of the strongly melanized hyphae of *S. japonica*, and progression of the discoloration was observed through the end of pollen dispersal season. At the season’s end, fungal development on the surface of the male strobili was visible without a microscope, because black stromata of the fungus were abundantly developed and had emerged from the opening microsporophylls (Fig. 3 F–H). The inoculum was successfully re-isolated only from treated male strobili. Conidial fructification was occasionally observed on the stromata six months after fungal application, and the conidia were most apparent under high humidity. Although immature perithecia were also found on the male strobili, ascospore production was not observed. Some conidial fructification observed in 2010 could still be observed on the trees at the end of 2012. However, infection by *S. japonica* did not appear to spread to other parts of the inoculated trees following initial treatment. The control trees were not infected by the fungus, and exhibited complete pollen release.

**Table 2:** Field trial of *Sydowia japonica* for prevention of pollen dispersal in Japanese cedar.

Treatment	Number of infected strobili/number of treated strobili in 2011 (field A)	Number of infected strobili/number of treated strobili in 2011 (field B)	Number of infected strobili/number of treated strobilii in 2012 (field C)	Total percent of prevented pollen dispersal (%)
FFPRI 411102*	18/20 ab***	14/20 ab	4/12 ab	69.2
FFPRI 411103	11/20 ab	9/20 ab	2/12 ab	42.3
FFPRI 411104	13/20 ab	10/20 ab	8/12 a	59.6
NITE P-757**	10/20 a	13/20 ab	5/12 ab	46
FFPRI 411105	13/20 ab	13/20 ab	5/12 ab	53.9
FFPRI 411084	N/A	N/A	7/12 ab	58.3
FFPRI 411087	17/20 ab	12/20 ab	9/12 a	73.1
FFPRI 411106	N/A	N/A	6/12 ab	50
FFPRI 411107	N/A	N/A	3/12 ab	25
FFPRI 411108	N/A	N/A	3/12 ab	25
FFPRI 411109	19/20 b	17/20 a	10/12 a	88.5
Control	0/20 c	0/20 b	0/12 b	0

* **FFPRI**: Forestry and Forest Products Research Institute, Ibaraki, Japan.

** **NITE**: Biological Resource Center, Chiba, Japan.

*** Numbers of each column followed by the same letter are not significantly different (Ryan’s test, P = 0.05).

### Behavior of S. japonica on Male Strobili with Stereomicroscope and SEM

**Figure 4:**
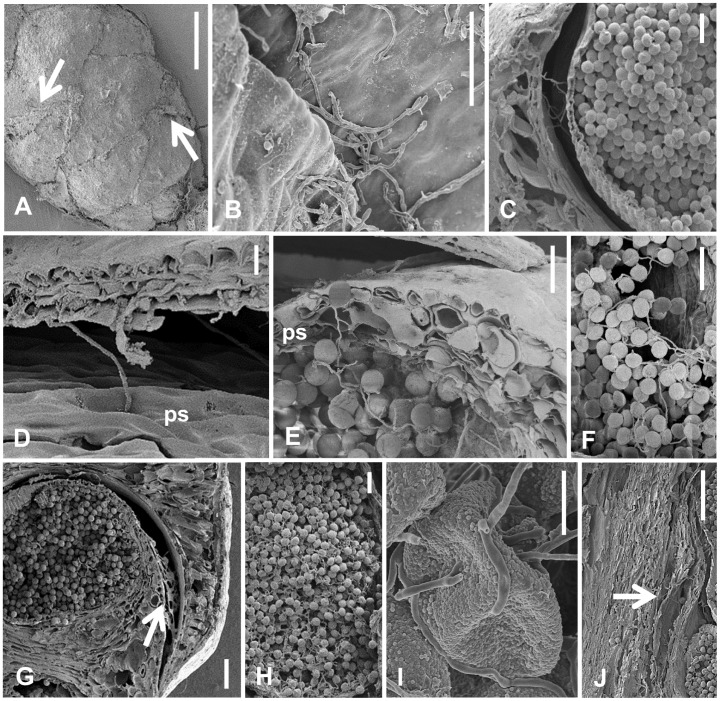
Scanning electron micrographs of male strobili of Japanese cedar treated with *Sydowia japonica*. A. Surface of a treated male strobilus and mycelia of *S. japonica* (arrows), about one month after treatment (beginning of December, 2011) (bar = 500 µm). B. Germinating hyphal fragment and conidia on surface of a male strobilus, about one month after treatment (bar = 50 µm). C. Hyphae on surface of a pollen sac, about one month after treatment (bar = 60 µm). D. Hyphae penetrating into a pollen sac (ps), about one month after treatment (bar = 10 µm). E. Hyphae penetrating into a pollen sac (ps) and pollen, about one month after treatment (bar = 50 µm). F. Hyphae penetrating into pollen, about one month after treatment (bar = 50 µm). G. Hyphal “net” (arrow) under a microsporophyll, about three months after treatment (middle of February, 2012) (bar = 100 µm). H. Pollen infected by hyphae of *S. japonica*, about six months after treatment (end of April, 2012) (bar = 50 µm). I. Pollen infected by hyphae of *S. japonica*, about six months after treatment (bar = 10 µm). J. Axis infected by hyphae of *S. japonica* (arrow), about six months after treatment (bar = 200 µm).

Behavior of *S. japonica* on male strobili in each month after treatment diagrammatically presented in order to clearly understand the infection process (Fig. 1). Observation of the fungal pathogenesis showed that hyphal fragments and conidia germinated on the surface of male strobili immediately following treatment, with germ tubes extending through the microsporophylls into the pollen sacs by one month post-infection (Fig. 4 A–C). The hyphae gradually covered or penetrated the pollen sacs, and a few hyphae started penetrating pollen grains themselves (Fig. 4 D–G). However, the growth of *S. japonica* was slow, and the fungus did not further penetrate during the winter season, from the beginning of December to the middle of February. By the middle of February, that is approximately one month before the start of the pollen dispersal season, the hyphae and conidia remaining on the male strobili regrew, and the all of the pollen grains were eventually infected (Fig. 3 B–E, H & Fig. 4 H, I). The infected pollen was never released, and male strobili including the infected pollen were eventually decomposed. Although the hyphae were often observed in central axial tissue of the strobili, the infection never extended to other parts of the infected trees, such as needles, stems, and buds (Fig. 4 J).

## Discussion

The efficacy of *S. japonica* for controlling the dispersal of Japanese cedar pollen was demonstrated in this study. To the best of our knowledge, this report represents the first instance of fungal biocontrol of pollen dispersal, although fungi have been developed for the biocontrol of pests, weeds, and plant diseases.

Our results indicated that any of eleven isolates of *S. japonica* collected around Japan were capable of controlling Japanese cedar pollen dispersal. Among them, the isolate collected from Kumamoto prefecture (FFPRI 411109), which represented the southernmost site of our collecting points, exhibited the highest efficacy (Table 2). Since our growth trial showed no significant difference between the Kumamoto isolate and the other isolates, we hypothesize that the Kumamoto isolate would be able to adapt to cedar forests conditions in other parts of Japan if the fungus were put into practical use as a biocontrol agent.

To understand the infection process of the fungus in the biocontrol of pollen dispersal, the pathogenesis of *S. japonica* after application to male strobili was observed by stereomicroscope and SEM. Our observations demonstrated that *S. japonica* did not intensively infect pollen before spring. Inoculation with *S. japonica* was conducted around the beginning of November, when the mature pollen in microspore stage appears and when *S. japonica* usually produces abundant spores. However, the initial growth of *S. japonica* on male strobili was impaired in the winter, presumably because of environmental conditions, notably low humidity and temperature. When growth of *S. japonica* resumed, around the middle of February, the mean temperature in our field was between 5 and 10°C, a temperature range that was adequate for fungal growth in our laboratory experiments. Thus, it is crucial to evaluate the ecological fitness of Japanese cedar and *S. japonica* under various environmental conditions in order to determine the optimal inoculation time.

Based on our observations, two properties of *S. japonica* hyphae appeared to have a great impact on the prevention of the pollen dispersal in our field trials. First, during the pollen dispersal season, *S. japonica* covered the pollen sacs and pollen with a “net” of hyphae. We speculate that this hyphal net presents a physical barrier to pollen dispersal. Second, hyphae were often observed entering the axes of the male strobili soon after pollen infection by *S. japonica.* This axis infection may impede stretching of the axes, preventing microsporophylls from opening wide enough for pollen dispersal.

Although the hyphae could infect central axial tissue of the strobili, the infection of *S*. *japonica* never extended to other parts of the Japanese cedar, such as needles, stems, and buds in our observation. According to Hirooka et al. [25], *S*. *japonica* was recorded on dead needles and buds of some specimens. However, these specimens were collected and identified by past researchers, and the all fungal fractifications on needles and buds identified as *S*. *japonica* were in fact immature black stromata based on our re-observation. So far many fungi that can produce the black fructification have been recorded from needles and buds of Japanese cedar such as *Mycosphaerella cryptomeriae* R. Shirai & Hara and *Plectosphaera cryptomeriae* (Hara) Tak. Kobay. [27]. We thus presume that the fungi were not *S*. *japonica*. However, additional sampling of *S*. *japonica* and continuous observation of treated Japanese cedar will be further confirmed in the conclusion.

After treatment, *S. japonica* could survive and keep suppressing the pollen dispersal for several years. Our observations showed that some male strobili treated in 2010 remained on the branches until 2012, and *S. japonica* kept producing conidia on those male strobili. In particular, conidial fructification, which contributes to the potential for fungal dispersal, often was observed on the infected male strobili, yielding conidial suspensions in large amounts; moreover, we occasionally observed such suspensions dripping from infected male strobili to non-infected male strobili in our field. The long-term potential of *S. japonica* control of pollen dispersal is supported by analogous properties of phylogenetically related fungi [25]. Specifically, *Hormonema carpetanum* Bills and *Endoconidioma populi* Tsuneda, Hambl. & Currah, both of which are regarded as relatives of *S. japonica*, are slow-growing, melanized, meristematic fungi typically observed on dead twigs, plant litter, or rock surfaces [28], [29]. These characteristics are considered to be necessary to tolerate a wide range of stresses [30]–[33]. Similarly, hyphae of *S. japonica* on Japanese cedar male strobili gradually melanize and can survive on the surface of male strobili and the opening microsporophylls even under severe winter conditions, including the low temperature, humidity, and nutrient availability observed in our field trials. Thus, *S. japonica* is expected to have strong viability on treated male strobili and long-term efficacy as a biocontrol agent.

Several species of fungi except *S*. *japonica* were rarely obtained from infected male strobili. Among these species, we occasionally observed that *Fusarium ciliatum* and *Zalerion* sp. specifically covered stroma of *S*. *japonica*. In general, *Clonostachys rosea* and *Trichoderma* sp., both of which were also found on infected male strobili, act as mycoparasitic fungi, while *F. ciliatum* and *Zalerion* sp. act as saprophytic fungi [34], [35]. Although parasitism of *F. ciliatum* and *Zalerion* sp. has not been examined, they might inhibit the growth of *S*. *japonica* and place obstacles on the road to use *S*. *japonica* as a biocontrol agent.

Efficacy of biocontrol agents is influenced by the methods used for production, formulation, delivery, and application of the organisms [36], [37]. For practical use of *S. japonica* in the field, several improvements of methods, especially for application methods and formulation, are needed. The granule inoculum containing hyphal fragments and conidia was used to evaluate the efficacy of *S. japonica* in our study. However, such an application technique would be too labor-intensive for practical use. Alternatively, radial sprays with fungal spores or hyphal fragments may replace the granule inoculum, and spraying by a helicopter in cedar forests may be one of the most ideal applications. While *S. japonica* can produce a certain quantity of conidia on artificial media according to Hirooka et al. [25], such growth is not abundant enough to use in extensive cedar forests. A suitable liquid culture producing plenty of conidia or hyphal fragments would have to be developed to provide conidial suspensions at levels sufficient for use as an inoculum. An optimal formulation for spraying would allow *S. japonica* to adapt to many different types of environments [37]. In general, fungal biocontrol agents, especially in the phyllosphere, require improved tolerance of desiccation [38], [39]. Although melanized hyphae of *S. japonica* appear to have strong viability as mentioned above, the fungus could not fully colonize under winter conditions of low humidity. Therefore, the improvement of desiccation tolerance will be needed to improve the efficacy of this proposed treatment.

Although we pointed out some traits of *S. japonica* as a biocontrol agent for controlling Japanese cedar pollen dispersal and some improvements required for utilization of *S. japonica*, the following factors necessary for successful use of biocontrol in nature would also need to be addressed: the epidemiology of the rhinitis to be controlled; the biology, ecology, and population dynamics of the biocontrol agent; and the interactions among these variables [36], [40]–[43]. The clarification of these factors could bring about the development of *S. japonica* for controlling Japanese pollen dispersal and relieving the symptoms of JCP.
